# Diagnostic Value of Whole-Body MRI Short Protocols in Bone Lesion Detection in Multiple Myeloma Patients

**DOI:** 10.3390/diagnostics11061053

**Published:** 2021-06-08

**Authors:** Davide Ippolito, Teresa Giandola, Cesare Maino, Davide Gandola, Maria Ragusi, Paolo Brambilla, Pietro Andrea Bonaffini, Sandro Sironi

**Affiliations:** 1 Department of Diagnostic Radiology, “San Gerardo” Hospital, Via Pergolesi 33, 20900 Monza, MB, Italy; teresagiandola@hotmail.it (T.G.); mainocesare@gmail.com (C.M.); gandolad@gmail.com (D.G.); maria.ragusi@gmail.com (M.R.); 2 School of Medicine, University of Milano-Bicocca, Via Cadore 48, 20900 Monza, MB, Italy; pa.bonaffini@gmail.com (P.A.B.); sandrosironi@libero.it (S.S.); 3 Department of Diagnostic Radiology, H Papa Giovanni XXIII, Piazza OMS 1, 24127 Bergamo, BG, Italy; pbrambilla@asst-pg23.it

**Keywords:** multiple myeloma, hematologic neoplasms, infiltration pattern, magnetic resonance imaging, diffusion-weighted imaging

## Abstract

The aim of the study is to evaluate the effectiveness of short whole-body magnetic resonance imaging (WBMRI) protocols for the overall assessment of bone marrow involvement in patients with multiple myeloma (MM), in comparison with standard whole-body MRI protocol. Patients with biopsy-proven MM, who underwent a WBMRI with full-body coverage (from vertex to feet) were retrospectively enrolled. WBMRI images were independently evaluated by two expert radiologists, in terms of infiltration patterns (normal, focal, diffuse, and combined), according to location (the whole skeleton was divided into six anatomic districts: skull, spine, sternum and ribs, upper limbs, pelvis and proximal two-thirds of the femur, remaining parts of lower limbs) and lytic lesions number (<5, 5–20, and >20). The majority of patients showed focal and combined infiltration patterns with bone lesions predominantly distributed in the spine and pelvis. As skull and lower limbs are less frequently involved by focal bone lesions, excluding them from the standard MRI protocol allows to obtain a shorter protocol, maintaining a good diagnostic value.

## 1. Introduction

Multiple myeloma (MM) is a plasma cell dyscrasia, characterized by proliferation and accumulation of monoclonal plasma cells [1]. MM is the second most common hematologic malignancy after non-Hodgkin lymphoma, and it is the most frequent cancer involving the skeleton, after metastasis [1]. Bone disease is one of the hallmarks of MM, considering that up to 80% of newly diagnosed cases present osteolytic lesions that lead to increased morbidity and mortality [2]. Therefore, a careful evaluation of the degree of skeletal involvement is of utmost importance in all patients suspected of MM, such as those with CRAB features (hypercalcemia, renal failure, anemia, and bone disease) or smoldering MM [2], as proposed by the revised International Myeloma Working Group (IMWG) criteria for the diagnosis of MM [2].

In this context, whole-body (WB) imaging techniques, such as computed tomography (CT) and fluorodeoxyglucose positron emission tomography (FDG-PET) acquired increased importance over the past few years. Among them, magnetic resonance imaging (MRI) has a high specificity and sensitivity in the detection of focal bone lesions and bone marrow infiltration, even before the mineralized bone has been destroyed [3,4,5,6]. According to recent literature, only lesions detected by MRI and FDG-PET are referred to as focal lesions and are different from lytic lesions detected with CT, where bone destruction has already taken place [6]. Whole-body MRI (WBMRI) is also the procedure of choice for evaluating painful complications and spinal cord compression in MM and the best noninvasive technique for differentiating neoplastic from osteoporotic vertebral fractures [7].

Although the clinical use of imaging modalities to diagnose MM is often influenced by the availability and affordability of different techniques, WBMRI remains the best imaging technique for evaluating bone marrow involvement [6]. WBMRI is recommended by the IMWG for all patients with suspected monoclonal gammopathy of undetermined significance (MGUS), smoldering MM, overt MM, and relapse in the case of negative or inconclusive CT [8,9,10]. Nevertheless, many issues need to be addressed to make WBMRI more widely accepted as the imaging modality of choice in all stages of MM patient management [2]. Its main limitations are in regard to availability, cost, technique standardization, radiologic expertise, and, above all, the image acquisition time, which makes the technique more cumbersome for patients, if compared to WBLDCT or PET/CT [11]. According to the recent guidelines published by Messiou et al., WBMRI acquisition for MM should include the vertex of the skull and the knees; if protocols are available, the lower extremities should also be shown in full [8]. However, a WMBRI including all bone segments from head to lower extremities can be challenging for patients, particularly those suffering bone pain.

In this setting, the present study aims to evaluate the effectiveness of shorter MRI protocols for the overall assessment of bone marrow involvement, according to different infiltration patterns in patients with MM, in comparison with standard whole-body MRI protocol.

## 2. Materials and Methods

Local Ethics Committee’s review of the protocol deemed that formal approval was not required owing to the retrospective, observational, and anonymous nature of this study.

We retrospectively evaluated all patients who had biopsy-proven MM diagnosed during the period from January 2017 to January 2020, who underwent WBMRI for disease staging.

Inclusion criteria were as follows: (1) age > 18 years, (2) MM diagnosis according to the International Myeloma Working Group, (3) WBMRI with full-body coverage (from vertex to feet).

Patients with nondiagnostic examinations due to artifacts or premature suspension of the scan were excluded.

### 2.1. WBMRI Protocol

**Figure 1:**
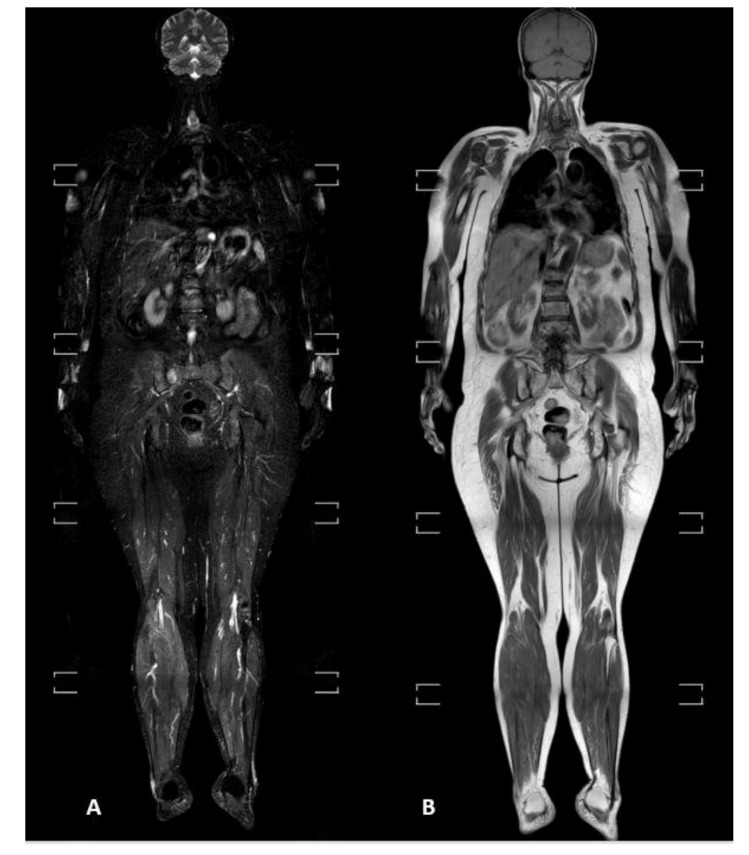
(**A**) T2-weighted short-tau inversion recovery (STIR) and (**B**) T1-weighted turbo spin-echo sequences acquired on the coronal plane from the skull vertex to feet.

**Figure 2:**
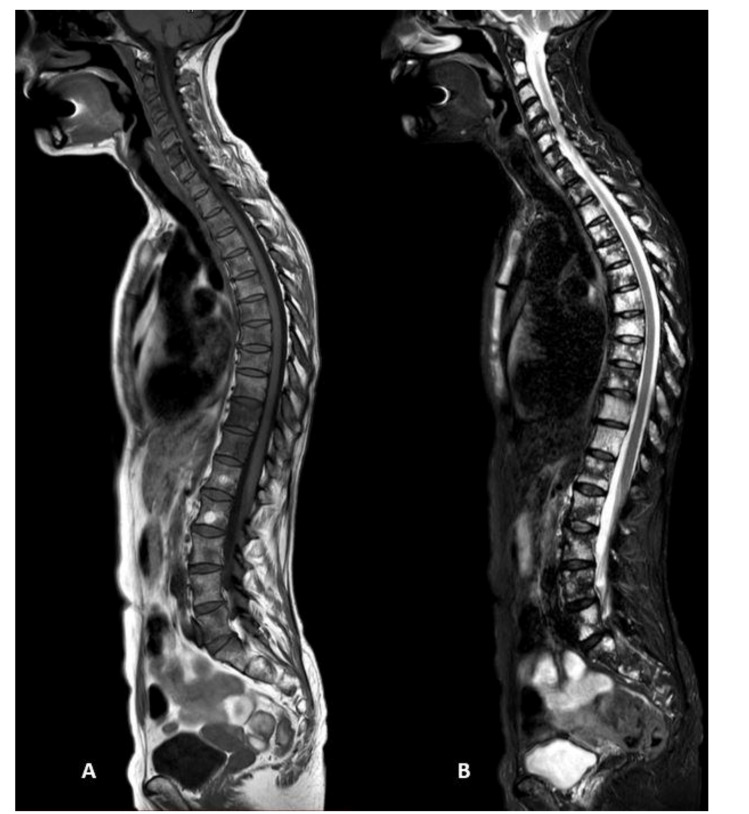
(**A**) T1-weighted turbo spin-echo and (**B**) T2-weighted short-tau inversion recovery (STIR) sequences of the spine acquired on the sagittal plane, showing multiple focal bone lesions hypointense on T1 sequences and hyperintense on T2 sequences, in a patient with focal infiltration pattern of multiple myeloma.

Whole-body MRI examinations were performed on a 1.5 T magnet (Ingenia, Philips, The Netherlands). The standard protocol included T1-weighted turbo spin-echo (TSE) and T2-weighted short-tau inversion recovery (STIR) sequences acquired on the coronal plane (from the skull vertex to the feet) (Figure 1) and on the sagittal plane for the spine (Figure 2). Diffusion-weighted imaging with background suppression (DWIBS) sequences were acquired on an axial plane with three b-values. All the study sequences were acquired during free breathing, with a slice thickness of 4 mm and a 1 mm gap. At the end of the study, every imaged district was merged using software integrated with the scanner, generating coronal whole-body T1, T2 STIR, spinal sagittal T1, T2 STIR, and DWIBS reconstructions.

The patient was positioned supine, headfirst, using two-phased body-array coils, inline for the examination of the thorax, abdomen, pelvic region, and upper and lower limbs, and one head-and-neck coil for the head and neck regions. All WBMRI studies were performed with the stepping-table movement technique.

### 2.2. Image Analysis

WBMRI images were independently evaluated by two radiologists, with 12 and 7 years of experience in MM and MRI imaging, to identify signs of bone involvement. Images were evaluated in terms of standard infiltration patterns, as normal, focal, diffuse, and combined.

The MRI diagnosis of bone marrow involvement was performed using mainly T1-weighted TSE and fat-suppressed sequences (STIR). Bone marrow pathological replacement, either focal or diffuse, leads to a signal intensity modification, namely a decrease on T1-weighted TSE and an increase on T2-weighted STIR images. The diffuse bone marrow involvement could also be seen as speckled and micronodular appearance (salt-and-pepper) with inhomogeneous bone marrow and interposition of fat islands [12].

The DWIBS sequences were used to confirm the type of infiltration pattern found on STIR and T1-TSE, considering that pathologic bone marrow usually exhibits restricted diffusion, with a higher signal on high b-value DWI compared to the very low signal of normal bone marrow [12].

The whole skeleton was divided into six anatomic districts: skull, spine, sternum and ribs, upper limbs, pelvis plus proximal two-thirds of the femur, and remaining parts of lower limbs. Therefore, all the typical focal bone lesions were recorded, according to the location, into two different categories: standard protocol (from vertex to feet) and short protocol (from vertex to thigh bone).

For each anatomic district, the two readers recorded the focal bone lesions according to their number as follows: less than 5, between 5–20, or more than 20 focal lesions.

The acquisition time of each sequence and the whole protocol was recorded.

The detailed MRI protocol is summarized in Table 1.

### 2.3. Statistical Analysis

Categorical variables were presented using median and IQR values, while continuous variables were given as mean ± standard deviation (SD). Intraclass correlation coefficients (ICC) and their 95% CI were calculated based on a mean-rating (k = 2), absolute-agreement, two-way mixed-effects model. Moreover, Kendall’s tau (τ) test was performed between ordinal variables: the tau correlation coefficient returns a value of 0 to 1, where 0 represents no relationship, 1 a perfect positive relationship. The 95% CIs were calculated using bootstrap with 500 iterations and random number seed 978. For both intertest qualitative reliability and inter-reader agreement, ICC values less than 0.5, between 0.5 and 0.75, between 0.75 and 0.9, and greater than 0.90 are indicative of poor, moderate, good, and excellent reliability, respectively.

For each anatomic district involved (skull, sternum and ribs, spine, upper limbs, pelvis, and lower limbs) a score value was assigned to categorize patients into three different groups: negative (score value = 0), low (1–3), and high (4–6) involvement. Areas under the receiver operating characteristic (AUROC) were calculated, as well as their 95% CIs, and were compared by using the DeLong test. All tests were two-sided, and the *p*-value of ≤0.05 was considered statistically significant. All the statistical analyses were performed by using IBM SPSS 26.0 (SPSS Incorporated, Chicago, IL, USA).

## 3. Results

### 3.1. Study Population

By applying the aforementioned inclusion and exclusion criteria, a total of 64 patients were analyzed. The majority was female (M/F = 34/29), with a mean age of 65 years (±10, range 41–84).

All MRI examinations were considered diagnostic from both readers, without any significant artifacts.

### 3.2. Agreement between Readers

The overall agreement between readers was very good regarding the pattern (κ = 0.954 (95% CIs: 0.885–1), *p* < 0.001). By grouping lesions’ distribution according to the anatomic district, the two readers showed a very good agreement for spine, skull, sternum and ribs, and pelvis (κ = 0.754, κ = 0.750, κ = 0.717, and κ = 0.727, respectively, all *p* < 0.001), and perfect or almost perfect agreement for upper and lower limbs (κ = 0.860 and κ = 1, respectively, all *p* < 0.05). All agreement values with 95% CIs are reported in Table 2.

Considering the good agreement between the two readers, only the results of the most experienced one were used for further analysis.

### 3.3. Radiological Pattern and Lesions’ Distribution

A total of 15/64 patients (23.4%) showed no lesions in all anatomic districts. Between patients with bone involvement (*n* = 49/64, 76.6%), the majority showed a focal pattern (*n* = 29/64, 59.2%), followed by combined (*n* = 16/64, 32.7%), and diffuse (*n* = 4/64, 8.1%) ones.

**Figure 3:**
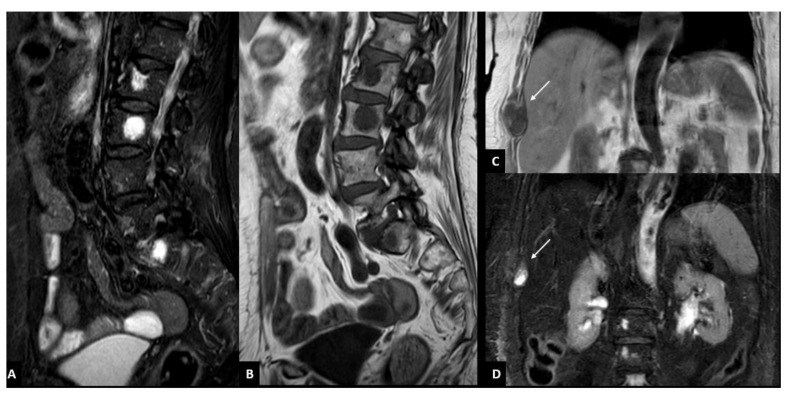
Focal infiltration pattern of multiple myeloma characterized by bone lesions of the spine (**A**,**B**) and the rib (**C**,**D**). Focal bone lesions of the spine are visible as a focal area of hypointensity on T1-weighted turbo spin-echo sequence (**A**) and of hyperintensity on T2-weighted short-tau inversion recovery (STIR) sequence (**B**) in L2, L3, and S1. Focal bone lesion of the rib is visible as focal area (arrow) of hypointensity on T1-weighted turbo spin-echo sequence (**C**) and of hyperintensity (arrow) on T2-weighted short-tau inversion recovery (STIR) sequence (**D**) in the costal angle of the ninth right rib.

Overall, the most common involved district was the spine (*n* = 40, 81.6) (Figure 3), followed by pelvis (*n* = 33, 67.4%), sternum and ribs (*n* = 23, 46.9%), upper limbs (*n* = 12, 24.5%), skull (*n* = 6, 12.3%), and lower limbs (*n* = 6, 12.3%). Table 3 summarizes the number of lesions and their distribution.

### 3.4. Standard vs. Short Protocols

To divide patients into different classes, we assigned one point for each considered district involved by focal lytic lesions, and the final score was computed by summing up each value. In this setting, the final score ranged between 0 and 6 points: “0” stands for patients negative for lytic lesions, while “6” indicates whole-body involvement.

**Figure 4:**
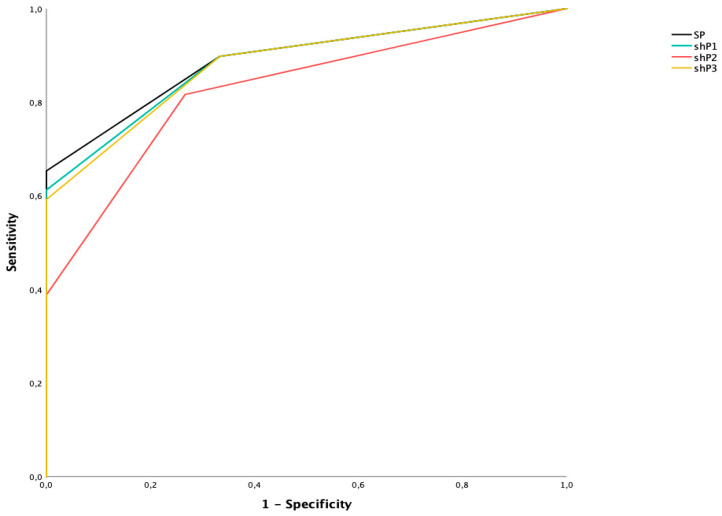
ROC curves of standard protocol (SP) and three different short protocols (shP1, shP2, shP3).

By using the standard protocol (Figure 4—SP), most patients showed a final score of 1 or 2 (*n* = 17/49 (26.6) and *n* = 14/49 (21.9), respectively), while only 18/49 (28.1) were categorized in classes 3, 4, and 5 (*n* = 8 (12.5), *n* = 7 (11.1), and *n* = 3 (4.7), respectively). The standard score presented a good sensitivity and specificity (89.8 (95% CIs: 77.8–96-6) and 66.7 (95% CIs: 38.4–88-2), respectively), and an overall good diagnostic accuracy (AUROC = 0.891; 95% CIs: 0.813–0.970).

By analyzing the short protocol (shP) removing the values regarding the lower limb involvement (Figure 4—shP1), the majority of patients showed a final score of 1 or 2 (*n* = 19/49 (29.7) and *n* = 13/49 (20.3), respectively), while only 17/49 (26.6) were categorized in classes 3, 4, and 5 (*n* = 9 (14.1), *n* = 6 (9.4), and *n* = 2 (3.1), respectively). Overall, this score reported a good sensitivity and specificity (89.8 (95% CIs: 77.8–96.6) and 66.7 (95% CIs: 38.4–88.2), respectively) with a slightly lower diagnostic accuracy (AUROC = 0.884; 95% CIs: 0.802–0.966).

By analyzing the short protocol removing the values regarding the skull (Figure 4—shP2), the majority of patients were classified in score 1 (*n* = 25/49, 39.1), followed by 2 (*n* = 11/49, 17.2), 3 and 4 (*n* = 7 (10.9) and *n* = 1 (1.6), respectively). This short protocol showed 81.7% sensitivity (95% CIs: 67.9–91.3) and 73.3% specificity (95% CIs: 44.9–92.2), with a good diagnostic accuracy (AUROC = 0.827; 95% CIs: 0.720–0.933).

Finally, excluding both skull and lower limb involvement (Figure 4—shP3), most patients reported a score of 1 (*n* = 20/49, 31.3), followed by 2, 3, and 4 (*n* = 14 (21.9), *n* = 8 (12.5), and *n* = 7 (10.9), respectively), showing 89.9% sensitivity (95% CIs: 77.8–96.6) and 66.7% specificity (95% CIs: 38.4–88.2), with a more than acceptable diagnostic accuracy (AUROC = 0.881; 95% CIs: 0.797–0.965).

The pairwise comparison between ROC curves showed no statistically significant differences between standard protocol (SP) and short protocols 1 and 3 (shP1 and shP3) (*p* = 0.209 and *p* = 0.141, respectively), while a significant difference was found in the comparison between SP and shP2 (*p* = 0.031).

### 3.5. Acquisition Time

SH, shP1, shP2, and shP3 mean acquisition times were 45 min and 20 s (±2′20″), 32 min and 10 s (±2′10″), 35 min and 40 s (±2′05″), and 28 min and 20 s (±2′15″), respectively.

## 4. Discussion

Whole-body MRI (WBMRI) is becoming increasingly relevant for the assessment of patients with MM, due to complete body coverage, excellent sensitivity for bone marrow involvement before or without bone destruction (i.e., in case of a diffuse pattern), and availability of advanced techniques such as diffusion-weighted imaging (DWI) and Dixon-based fat-fraction evaluation [13,14,15].

According to the current literature, WBMRI is considered a valid option for bone marrow imaging by the European Society for Medical Oncology guidelines, and in the UK, it is recommended as first-line imaging for all patients with a suspected new diagnosis of myeloma [16,17].

Furthermore, the European Myeloma Network guidelines also recommend WBMRI in asymptomatic SMM (smoldering multiple myeloma) patients with no detectable lytic disease on CT [18], at initial diagnosis and then yearly [19].

In our series, WBMRI was used to analyze the skeletal and bone marrow involvement in 64 patients with a diagnosis of MM, which was classified into four different infiltration patterns (normal, focal, diffuse, and combined): The majority of patients showed skeletal involvement by bone lytic lesions with both focal and combined patterns, in line with the literature [20]. Only a small number of patients, instead, presented a diffuse bone marrow infiltration (8%), with no evidence of focal lytic lesions. Finally, 23% of enrolled patients presented a normal pattern without any bone marrow involvement.

When analyzing the anatomic distribution of bone marrow involvement in the focal and combined pattern of disease, our results showed that the lytic lesions were predominantly distributed in the spine and the pelvis (including the proximal two-thirds of the femur), in line with previous papers [21,22,23]. Moreover, the analysis concerning the number of bone lesions, according to the Durie and Salmon staging system [24], highlighted that the majority of the lesions was localized in the anatomic districts previously described. In 40 patients with bone lesions in the spine, exactly half of them presented >5 lesions (particularly, 5 patients had >20 focal bone lesions). In 33 patients with bone lesions in the pelvis and the proximal part of femurs, 40% of them showed >5 lesions.

The anatomic districts less frequently involved were the skull, the lower limbs (from the distal part of the femurs to the feet), and the upper limbs. Overall, 90.6% of patients did not show any focal bone lesion in the skull or in the lower limbs. Considering the number of lesions recorded, in the skull, no patients showed >20 lesions and only 3% of patients had between 5 and 20 focal lesions.

When analyzing the focal lesions in the lower limbs, the total amount of lesions was considerably lower; in fact, no patients with focal involvement of the lower limbs presented >5 lesions in this anatomic district. The obtained results highlighted that most patients with bone lesions in the skull and lower limbs presented >20 focal lesions disseminated in other anatomic districts, being classified in stage III of the Durie and Salmon staging system.

Therefore, we may assume that bone lesion detection in these two anatomic districts can have negligible relevance for staging, prognosis, and treatment evaluation.

After a careful evaluation of the results, we tried to optimize the standard protocol of WBMRI (from vertex to feet), by shortening it, maintaining adequate sensitivity, specificity, and diagnostic accuracy. Over the past decade, there has been an increased interest in short MRI protocols, aimed to preserve the diagnostic potential while reducing the scanning time and the potential discomfort.

In the simulated short protocols, we excluded from the standard protocol the two anatomic districts less involved by bone lesions: skull and lower limbs (from the distal third of the femur to the feet).

The obtained results showed that the short MRI protocols excluding the skull (shP2) or the lower limbs (shP1) presented in both cases a good sensitivity and specificity (82% and 73%, and 90% and 67%, respectively) and a sufficient diagnostic accuracy (88.4% and 82.7%, respectively) if compared with a standard protocol (SP). Moreover, the shortest MRI protocol, which excludes both skull and lower limbs (shP3), along with good sensitivity and specificity (90% and 67%, respectively), reached a more than acceptable diagnostic accuracy (88.1%).

Results in terms of accuracy did not show a statistically significant difference among the MRI short protocols proposed (*p* > 0.05), highlighting the sufficient efficacy of the shortest protocol (shP3), which might be considered in the clinical practice, in particular for the follow-up.

Excluding both the abovementioned anatomic districts (skull and lower limbs) the MRI protocol allows to save about 15 min in comparison to the standard protocol, thus representing a more acceptable and comfortable diagnostic option for patients with multiple myeloma.

Some limitations should be noted, first of all, the retrospective design of the study, and secondly, the small size of the cohort studied, due to the strict inclusion criteria.

## 5. Conclusions

In conclusion, a short WBMRI protocol, excluding the anatomical regions of the skull and the lower extremities, could represent a reliable diagnostic imaging tool for the evaluation of MM patients, shortening the duration of MRI examination, maintaining an overall good sensitivity, specificity, and diagnostic accuracy and also reducing the patient’s potential discomfort.

## Figures and Tables

**Table 1:** Detailed scanning parameters for all sequences.

Sequence	District	TE (ms)	TR (ms)	NSA	DFOV (mm)	Voxel Size (mm)	Thickness (mm)	Time (s)
**T1 TSE Coronal**	Head	15	922	1	365	1.16 × 1.46	6	50
	Thorax	15	922	1	365	1.16 × 1.46	6	50
	Abdomen	15	922	1	365	1.16 × 1.46	6	50
	Lower limb	15	922	1	365	1.16 × 1.46	6	39
	Feet	15	922	1	365	1.16 × 1.46	6	39
**T2 STIR TSE Coronal**	Head	60	8704	1	365	1.25 × 1.82	6	87
	Abdomen	60	8704	1	365	1.25 × 1.82	6	71
	Upper limb	60	8704	1	365	1.25 × 1.82	6	87
	Lower limb	60	8704	1	365	1.25 × 1.82	6	90
	Feet	60	8704	1	365	1.25 × 1.82	6	90
**T1 TSE Sagittal**	Cervical	7.4	408	3	270	0.90 × 1.15	3.5	300
	Thorax	7.4	408	2	270	0.90 × 1.15	3.5	188
**T2 STIR TSE Sagittal**	Cervical	60	2533	2	270	0.90 × 1.25	3.5	225
	Thorax	60	2533	2	270	0.90 × 1.25	3.5	167
**DWIBS Axial**	Head	66	6421	2	520	5.00 × 4.98	6	135
	Abdomen	66	6421	2	520	5.00 × 4.98	6	135
	Upper limb	66	6421	2	520	5.00 × 4.98	6	135
	Lower limb	66	6421	2	520	5.00 × 4.98	6	135
	Feet	66	6421	2	520	5.00 × 4.98	6	135
	Cervical	66	6421	2	520	5.00 × 4.98	6	135
	Thorax	66	6421	2	520	5.00 × 4.98	6	135

TSE: turbo spin echo, STIR: short-TI inversion recovery, DWIBS: diffusion-weighted whole-body imaging with background body signal suppression, TE: echo time, TR: repetition time, NSA: number of signals averaged, DFOV: display field of view.

**Table 2:** Agreement between the two readers regarding MM pattern and lesions numbers according to anatomic districts (skull, sternum and ribs, spine, upper limbs, pelvis, and lower limbs). The 95% CIs were computed by bootstrap.

N = 64	Agreement (κ; 95% CIs)	*p*-Value	τ-Value (95% CIs)	*p*-Value
Pattern *	0.954 (0.885–1)	<0.0001	0.958 (0.894–1)	<0.0001
Skull ^	0.750 (0.329–1)	<0.0001	0.745 (0.368–1)	0.014
Sternum and ribs ^	0.717 (0.541–0.861)	<0.0001	0.820 (0.688–0.935)	<0.0001
Spine ^	0.754 (0.621–0.878)	<0.0001	0.881 (0.810–0.942)	
Upper limbs ^	0.860 (0.662–1)	<0.0001	0.856 (0.670–1)	<0.0001
Pelvis ^	0.727 (0.577–0.862)	<0.0001	0.855 (0.757–0.935)	<0.0001
Lower limbs ^	1 (1–1)	<0.0001	1 (1–1)	0.004

* Grouped as negative findings, focal, diffuse, combined. ^ Grouped as no lesions, <5 lesions, 5–20 lesions, >20 lesions.

**Table 3:** Lesions’ distribution according to anatomical district and number. The most commonly involved anatomic district was the spine, followed by pelvis and sternum, and ribs.

N = 64	Negative Findings	Positive Findings		
		<5 Lesions	5–20 Lesions	>20 Lesions
Skull (*n*, %)	58/64 (90.6)	4 (6.3)	2 (3.1)	0 (0)
Sternum and ribs (*n*, %)	41/64 (64.0)	12 (18.8)	9 (14.1)	2 (3.1)
Spine (*n*, %)	24/64 (37.5)	20 (31.2)	15 (23.5)	5 (7.8)
Upper limbs (*n*, %)	52/64 (81.2)	7 (10.9)	4 (6.3)	1 (1.6)
Pelvis (*n*, %)	31/64 (48.4)	20 (31.3)	11 (17.2)	2 (3.1)
Lower limbs (*n*, %)	58/64 (90.6)	6 (9.4)	0	0
